# The rapid “teabag” method for high-end purification of membrane proteins

**DOI:** 10.1038/s41598-020-73285-9

**Published:** 2020-09-30

**Authors:** Jenny Hering, Julie Winkel Missel, Liying Zhang, Anders Gunnarsson, Marie Castaldo, Per Amstrup Pedersen, Margareta Ek, Pontus Gourdon, Harm Jan Snijder

**Affiliations:** 1 Structure, Biophysics and FBLG, Discovery Sciences, R&D, AstraZeneca, Gothenburg, Sweden; 2 Department of Chemistry and Molecular Biology, University of Gothenburg, Gothenburg, Sweden; 3 Department of Biomedical Sciences, University of Copenhagen, Copenhagen, Denmark; 4 Discovery Biology, Discovery Sciences, R&D, AstraZeneca, Gothenburg, Sweden; 5 Department of Biology, University of Copenhagen, Copenhagen, Denmark; 6 Department of Experimental Medical Science, Lund University, Lund, Sweden

**Keywords:** Biochemistry, Proteins

## Abstract

Overproduction and purification of membrane proteins are generally challenging and time-consuming procedures due to low expression levels, misfolding, and low stability once extracted from the membrane. Reducing processing steps and shortening the timespan for purification represent attractive approaches to overcome some of these challenges. We have therefore compared a fast “teabag” purification method with conventional purification for five different membrane proteins (MraY, AQP10, ClC-1, PAR2 and KCC2). Notably, this new approach reduces the purification time significantly, and the quality of the purified membrane proteins is equal to or exceeds conventional methods as assessed by size exclusion chromatography, SDS-PAGE and downstream applications such as ITC, crystallization and cryo-EM. Furthermore, the method is scalable, applicable to a range of affinity resins and allows for parallelization. Consequently, the technique has the potential to substantially simplify purification efforts of membrane proteins in basic and applied sciences.

## Introduction

Membrane proteins play a crucial role in many essential physiological functions in all organisms—including signal transduction, molecular recognition and ion transport to control cellular responses in health and disease states^1^. Modulating these pathways is attractive as therapeutic intervention, and around 50% of current drugs on the market target membrane proteins^2,3^, demonstrating the pharmacological importance of this protein class. However, membrane proteins are challenging to express and purify in functional form. Thus, drug discovery approaches for membrane proteins rely to large extent on phenotypic screening or target directed cell-based screening, while structure-based drug discovery, biochemical high-throughput screening and biophysical applications remain demanding.

Multiple hurdles exist for production of high quality membrane protein samples: natural abundance is typically low, and recombinant overexpression often leads to aggregation, misfolding or toxicity issues^4^, and as consequence levels of correctly inserted membrane proteins are often modest. Ironically, the next stumbling block arises as correctly inserted membrane protein targets require to be extracted from their membrane environment for purification and downstream applications, while maintaining their active form^5^. Once extracted, membrane proteins are often aggregation-prone and show reduced stability and activity.

A wide range of strategies are emerging to address stability issues of extracted membrane proteins including novel detergents^6,7^ and polymers^8,9^, methods for rapid screening of buffer conditions and solubilization agents^10–12^, use of ligands or lipids to increase stability^13,14^, and mutagenesis approaches^15,16^. Despite these measures, short purification procedures (minimizing the time between membrane protein extraction and the application) are essential to maximize the potential outcome in i.e. biophysics, structure determination and biochemical assays.

**Figure 1:**
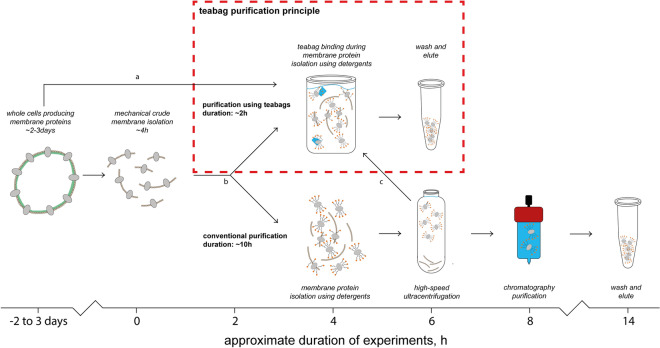
Overview and comparison of the conventional and teabag purification. Considerable time is saved with maintained protein quality using the purification procedures presented in this work. Teabag purification can be applied at various stages indicated by arrows a, b, and c, respectively for solubilization from whole cells, solubilization from membranes, and for purification after solubilization.

Previously, we have developed a “teabag” purification system for fast and easy purification of secreted proteins^17^. For “teabag” purification, the affinity resin is contained in a porous bag, which can be incubated in growth-broth containing cells or unclarified lysate, to efficiently capture target proteins. In this work, we have evaluated the “teabag” purification system for five different membrane proteins: the *Clostridium bolteae* MraY enzyme involved in bacterial cell wall synthesis that has been heterologously expressed in *Escherichia coli*, the human AQP10 water channel and the human chloride ion conducting ClC-1 channel (the E232A mutant form targeting a central gate-residue) expressed in yeast *Saccharomyces cerevisiae*, as well as the human PAR2 GPCR, and *Monodelphis domestica* KCC2 ion channel both heterologously expressed in insect cells. For the selected targets, purification results in purity and quality of samples that are identical to or exceed the established strategies, while enabling a significantly reduced purification time (Fig. 1). We further show that “teabag” purified membrane proteins are compatible with downstream applications, such as enzymatic assays, reconstitution into liposomes, ITC, crystallization and cryo-EM.

## Results

### Purity, yield and chromatographic behavior

**Figure 2:**
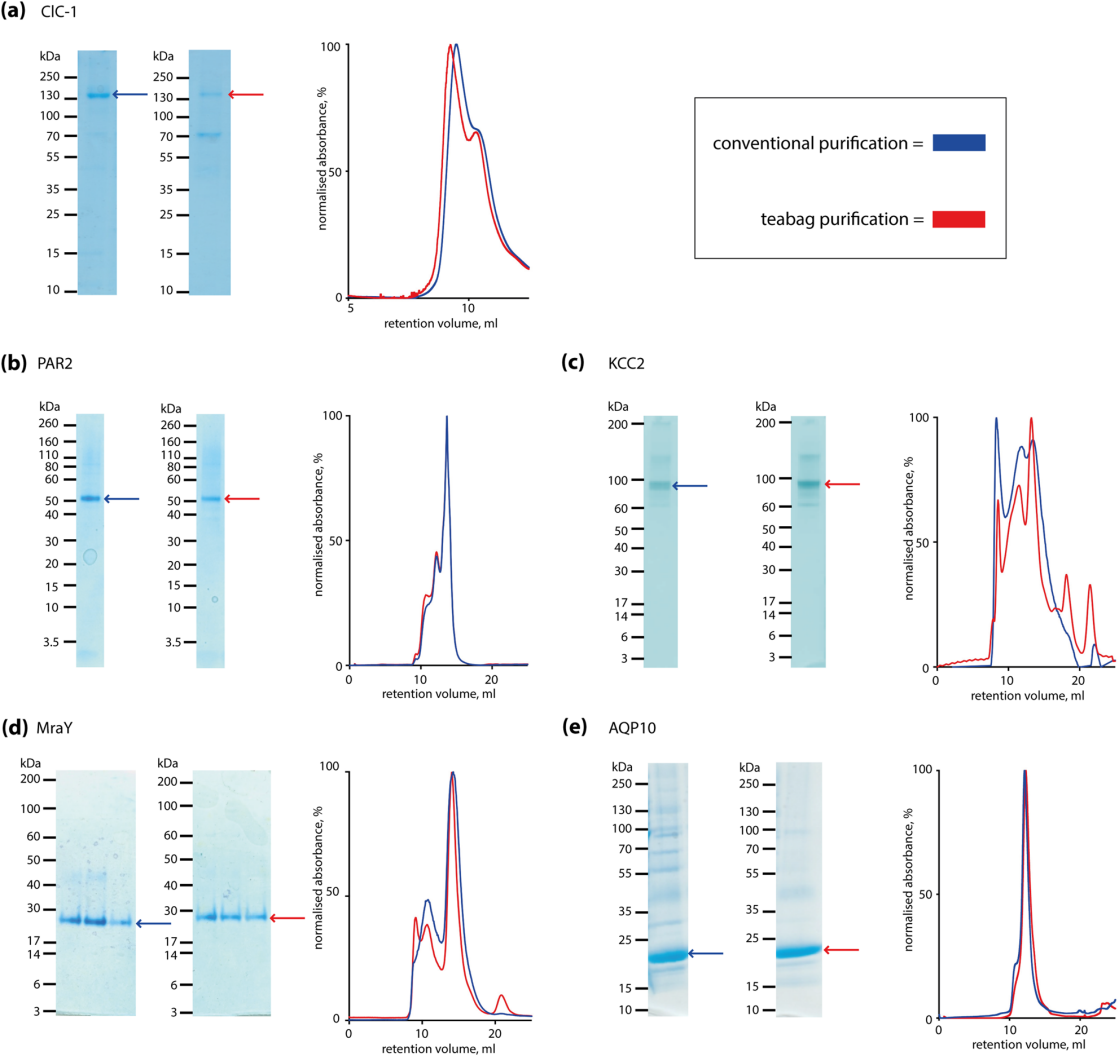
Comparison of conventional and teabag purification of five different membrane proteins. Each panel shows the results from side-by-side comparison of conventional and teabag purifications, respectively. SDS-PAGE with conventional (left) and teabag (right) purification, and SEC profiles with conventional purification (blue) and teabag purification (red). (**a**) Human ClC-1 chloride channel; (**b**) human PAR2; (**c**) *Monodelphis domestica* KCC2; (**d**) *Clostridium bolteae* MraY; (**e**) human AQP10. For each pairs of SDS-PAGE images, comparable amounts of protein have been loaded and the brightness and contrast is equal and applied across the entire image, non-cropped SDS-PAGE images are provided in the Supplementary Material.

In order to evaluate the capability of the teabag system, we have expressed different membrane protein targets and purified each of them side-by-side with teabags and conventional purification methods. For adequate comparison, conventional and teabag purifications were performed in parallel using the same cell or membrane preparations. The purity of the proteins visualized by SDS-PAGE and size exclusion elution profiles are shown in Fig. 2, while yield, resin and purification times are listed in Table 1.

**Table 1:** Summary of purification results.

Target	Expression host	Teabag purification resin and method	Yield (mg/L)	Conventional yield (mg/L)	Purification time teabag/conventional (hrs)	QC & application
MraY	*E. coli*	IMAC Talon	0.7	1.1	16/24	SEC, enzymatic assay, nanoDSF, ITC
ClC-1	*S. cerevisiae*	IMAC Ni–NTA	0.8	1.0	8/14	SEC, negative staining
hAQP10	*S. cerevisiae*	IMAC Ni–NTA	1.2	1.8	8/14	SEC, proteoliposomes, crystallization
PAR2	Sf9 insect cells	IMAC Ni–NTA	0.6	0.7	7/10	SEC
KCC2	Sf9 insect cells	FLAG	0.5–1.0	1.5	6/12	SEC, ligand binding by SPR
KCC2	Sf9 insect cells	FLAG insoluble fraction not removed	0.5–1.0	1.5	6/12	SEC, ligand binding by SPR

With respect to purity, only minor differences are observed between the purification methods. The SDS-PAGE in Fig. 2a shows that for teabag purified ClC-1 an additional lower molecular weight band occurs that is absent after conventional purification. For PAR2 and KCC2, the purity as judged by SDS-PAGE (Fig. 2b,c) is equal between the purification methods. However, for both MraY (Fig. 2d) and hAQP10 (Fig. 2e), the teabag material appears to be more pure.

While protein purity is an important criterion, size-exclusion profiles are considered the golden standard for membrane protein quality control. The ClC-1 E232A variant displays a large void peak (Fig. 2a), with a shoulder at 10 mL corresponding to the fraction of interest. For the teabag purified ClC-1, this shoulder is better resolved from the void peak and the ratio peak-of-interest/void is higher as compared to the conventionally purified ClC-1. In case of MraY (Fig. 2d) and hAQP10 (Fig. 2e) the size exclusion profiles show sharper protein peaks, indicating improved sample quality. For KCC2 (Fig. 2c), size exclusion profiles also indicate quality improvement when using teabag purification. Conventional purification of KCC2 results in three large SEC peaks at 8 mL, 12 mL and 13.6 mL, respectively. These present aggregated KCC2 (8 mL), a non-ligand binding competent form (12 mL) and ligand binding competent KCC2 (13.6 mL). For teabag purified KCC2, the SEC profile shows smaller peaks at 8 and 12 mL, while the 13.6 mL feature is sharper and higher. Thus, the relative fraction of ligand binding competent KCC2 after teabag purification is substantially larger.

As listed in Table 1, the overall yield for the tested targets was lower for the teabag method in comparison to the conventional purification and varied for the different membrane proteins. Nevertheless, yields in the mg range were obtained in all cases, sufficient for downstream applications. The teabag purification was significantly faster for all tested targets as listed in Table 1. In case of KCC2, the purification time was considerably shortened from approximately 12 to 6 h with the teabag purification procedure. Similarly, the other protocols were shortened so that the total purification time became well manageable within a single working day.

### Downstream applications

**Figure 3:**
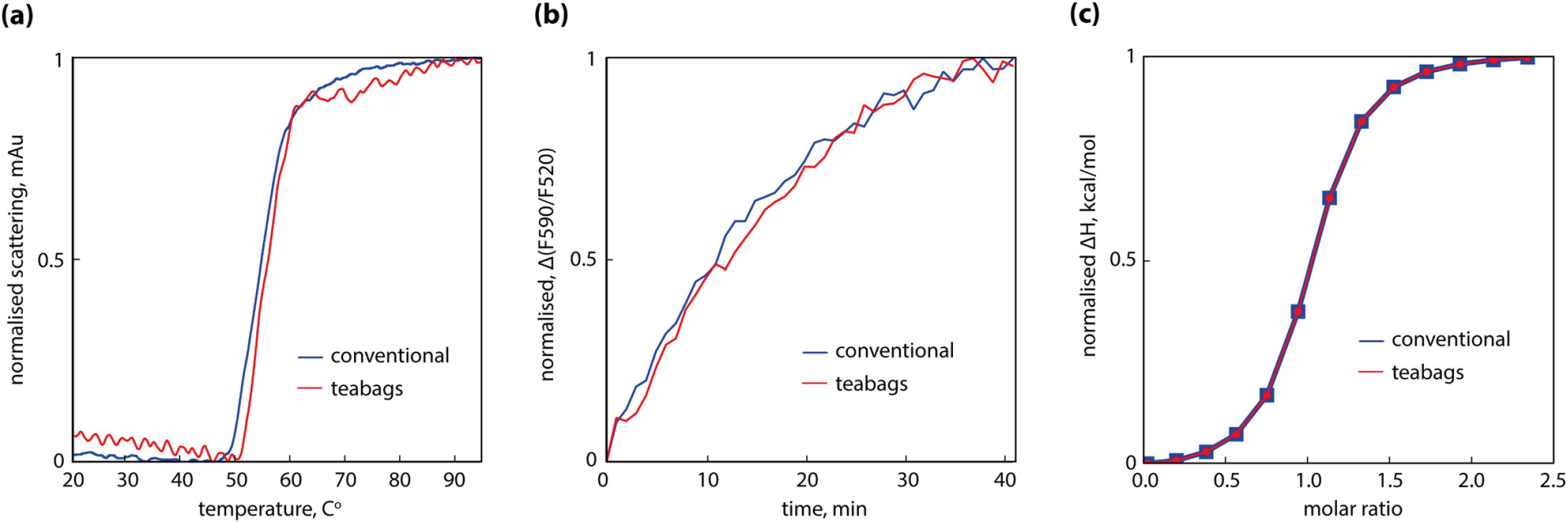
Quality assessment of purified MraY and comparison between conventional (blue) and teabag purification (red). (**a**) Nano DSF thermal stability; (**b**) FRET based activity assay; (**c**) Tunicamycin binding as measured by ITC.

In order to further evaluate quality, functionality and stability of the teabag samples, we have analyzed selected targets in functional assays in comparison to conventionally purified proteins and assessed their usability in downstream applications. In case of MraY, we have determined its thermal stability and enzymatic activity from teabag material using a FRET-based activity assay^18,19^. We observed membrane protein samples of MraY showing equal specific enzymatic activity (Fig. 3b), and a melting curve that was identical to samples obtained with the conventional purification (Fig. 3a). The teabag samples were further used in ITC experiments in order to determine the dissociation constant K_d_ for the MraY inhibitor tunicamycin with a measured K_d_ value of 233 nM, identical to conventional purified MraY (Fig. 3c). In addition, we reproduced initial crystal hits with mg-amounts of MraY purified with the teabag system.

**Figure 4:**
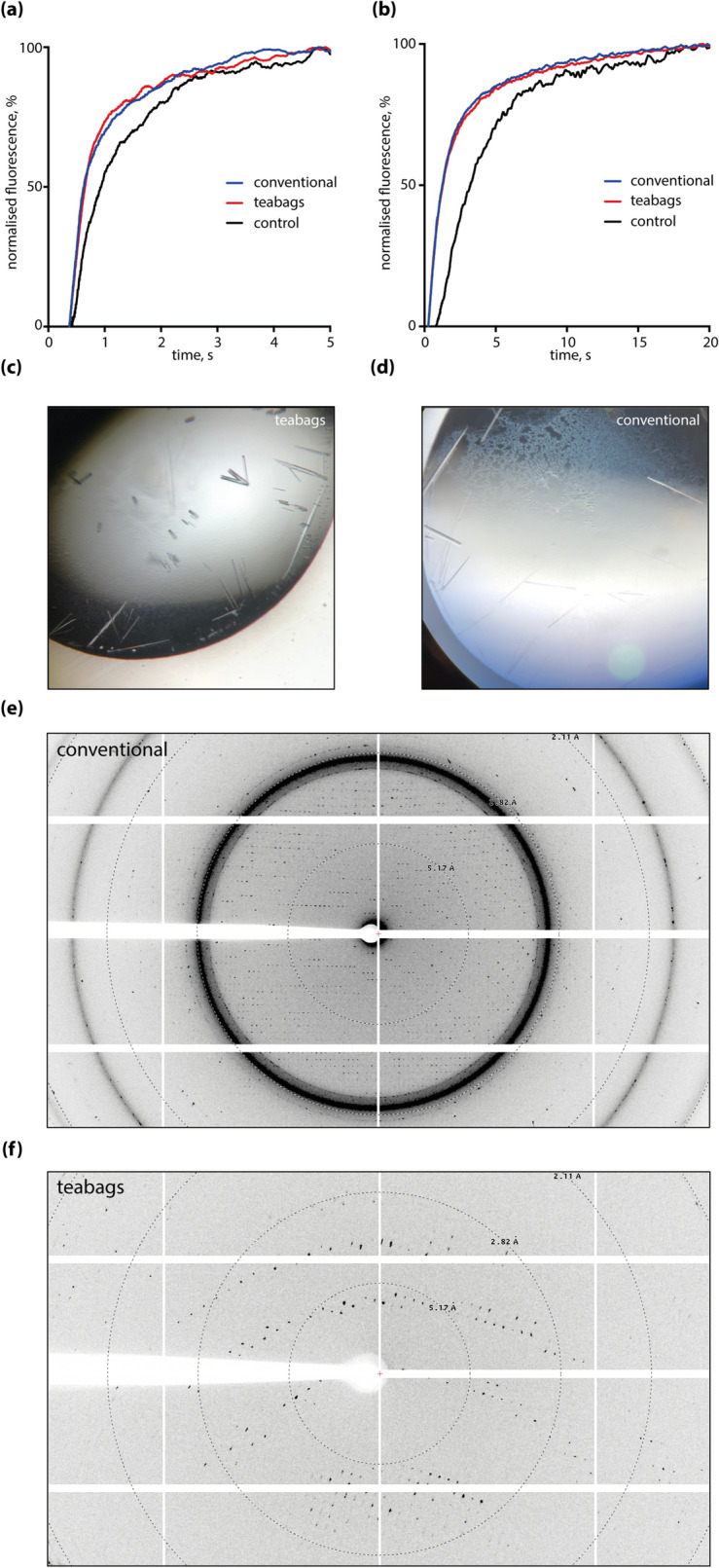
Quality assessment of purified hAQP10 using a liposome assay and crystallization. (**a**) Water efflux assay of hAQP10 reconstituted proteoliposomes. Almost identical traces of conventional (blue) and teabag (red) purified samples, using empty liposomes as control (black); (**b**) glycerol efflux assay of hAQP10 reconstituted proteoliposomes. Almost identical traces of conventional (blue) and teabag (red) purified samples, using empty liposomes as control (black); (**c**) crystals produced from teabag purified hAQP10, showing rod-shaped crystal morphology; (**d**) crystals produced from conventionally purified hAQP10, showing needle-shaped crystal morphology; (**e**) diffraction pattern of conventionally purified hAQP10, showing a diffraction to approximately 2 Å; (**f**) diffraction pattern of teabag purified hAQP10, showing a diffraction to approximately 2 Å.

Following size exclusion of hAQP10, we performed crystallization trials as previously reported^20^ along with reconstitution experiments into liposomes for activity measurements. Glycerol and water flux through reconstituted hAQP10 purified using teabag are in agreement with conventionally purified hAQP10 (Fig. 4a,b). Crystallization experiments yielded large rod-shaped crystals (Fig. 4c) with a slightly different morphology compared to the thin needle shaped hAQP10 crystals when using the classical purification procedure (Fig. 4d). This variance may be caused by the changed purity of the hAQP10 sample, possibly altering the lipid and/or detergent content of the sample. The diffraction of both types of crystals yielded diffraction to 2.0–2.5 Å (Fig. 4e,f), like what has already been reported^20^.

**Figure 5:**
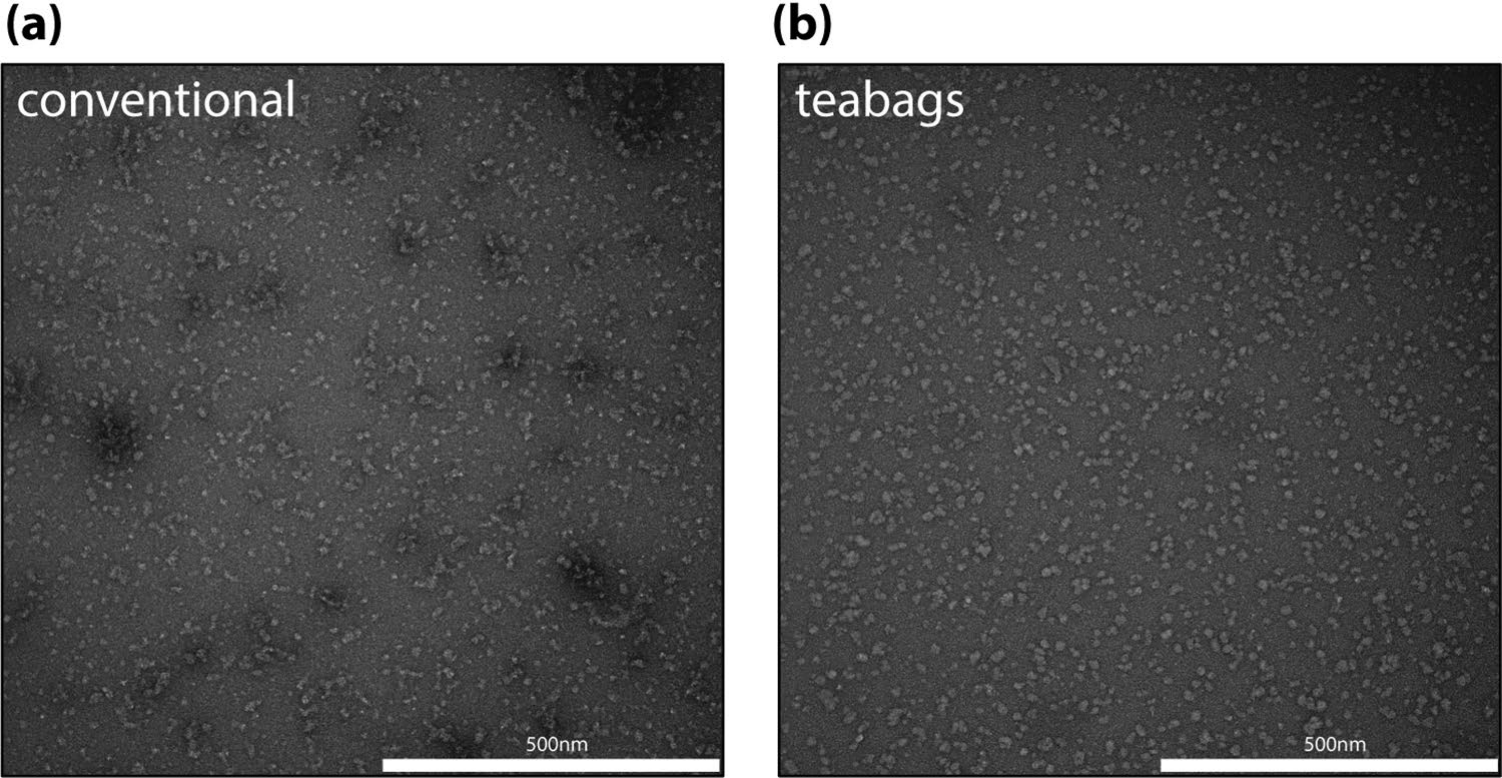
Quality assessment of purified ClC-1 using negative staining EM. (**a**) Conventionally purified ClC-1, showing higher degree of aggregation and increased heterogeneity in particle size and distribution; (**b**) teabag purified ClC-1, showing evenly distributed particles.

We have analyzed E232A ClC-1 purified protein samples using electron microscopy. Negative stained electron micrographs revealed that the particles from the teabag samples were evenly distributed (as for the previously reported wild-type protein^21^), while the particles from the conventional purification were more heterogeneous both in terms of particle shape and particle distribution (Fig. 5a,b). Thus, despite a slightly lower purity judged by SDS-PAGE (Fig. 1a), the teabag purified ClC-1 may be the preferred specimen for cryo-EM procedures due to the higher homogeneity, although the conventionally purified wild-type ClC-1 samples already has resulted in a cryo-EM structure ^21^.

**Figure 6:**
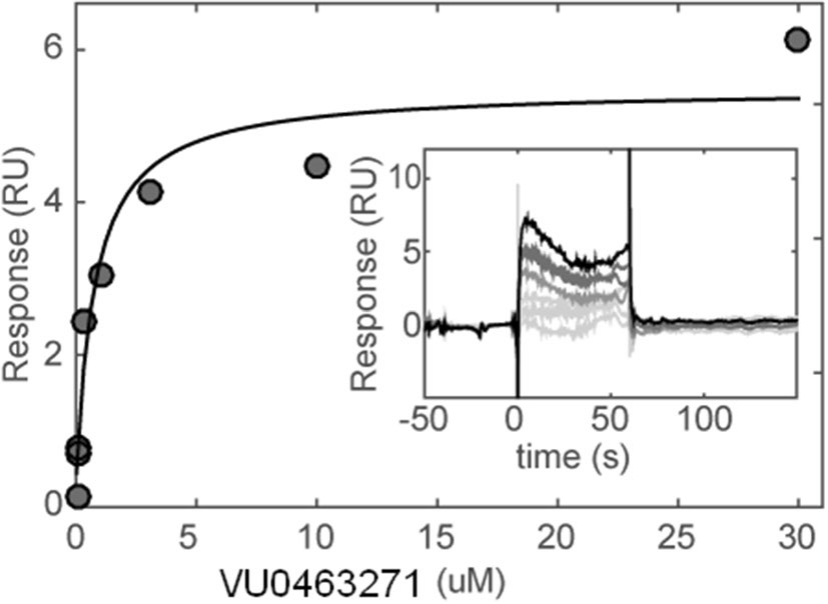
Direct ligand binding assays of the teabag purified KCC2 by surface plasmon resonance (SPR). Concentration–response and corresponding Langmuir steady state fit of VU0463271 injected onto covalently immobilized KCC2. Inset: sensorgram of binding of (from bottom to top) 0, 0.1, 0.3, 1, 3, 10, 30 µM compound.

For KCC2 direct ligand binding was measured using surface plasmon resonance (SPR). Teabag purified KCC2 showed saturated binding of compound VU0463271^22^ with a K_d_ of 750 ± 250 nM (Fig. 6) and the data were comparable to SPR binding data for conventionally purified KCC2 (data not shown).

## Discussion

Despite recent advances in the field, the production and purification of membrane proteins in their active forms remain a major challenge, and consequently e.g. high-resolution structures of membrane proteins are significantly under-represented in the structural protein database PDB^23^. To address such membrane protein purification issues, we have successfully applied the teabag purification method to membrane protein targets. The technique is straightforward, inexpensive and significantly shortens the purification procedure, and established protocols can easily be adapted to teabag purification.

Using the teabag system, we have purified five membrane proteins, covering different target families, expression systems and detergent classes. We purified all examined membrane proteins to high quality using the teabag system on a substantially shorter time-scale compared to conventional purification methods. Despite subtle effects on the overall yield for teabag purification, we obtained sufficient amounts of purified membrane proteins for use in crystallization trials and ITC experiments (hAQP10 and MraY), which are techniques that consume mg-amounts of sample. Furthermore, lower purification yields may well be acceptable, when this is accompanied by improved quality of the membrane protein. This is particularly demonstrated for ClC-1, where negative staining EM after size exclusion verified a more homogeneous and better quality of the teabag purified sample.

Analysis of the size exclusion profiles for all tested targets suggests that teabag purification results in equal or better quality of the membrane proteins as shown by smaller fractions of the void and sharper elution peaks, which indicates less aggregation material in the teabag samples. In agreement with this notion, the size exclusion profile of the teabag purified E232A ClC-1 variant indicates improved stability of the sample as compared to the conventionally purified sample.

The teabag system seems to enable a milder purification for membrane proteins, while the underlying mechanisms are not fully clear to the authors. The shorter purification times could be the prime cause for the observed improvement in quality and stability of some membrane proteins. Additionally, more delicate purification conditions could contribute to improved quality; during teabag purification, the immobilized membrane proteins are maintained under gentle stirring without applying any pressure or force, in contrast to standard purification using gravity flow columns and/or automated HPLC systems. The teabag purification may also shift the equilibrium during solubilization, reducing the pool available for affinity-binding of solubilized protein, and/or prevent aggregation, as immobilization of the targets on the purification resin permits rapid separation of the target protein from cell debris, filamentous and aggregated material that may otherwise form nuclei for further aggregation. Another aspect is the critical effect of lipids on the functionality of membrane proteins. Recent studies suggest slow kinetics for de-lipidation of membrane proteins relative to the faster solubilization process^24^. This may imply that the samples from conventional purification can be delipidated to a larger extent compared to the teabag samples due to the longer purification time. Removal of lipids could result in reduced quality and poorer behavior of the membrane protein preparation. Although these hypotheses are speculative, understanding the basis for improved purification and kinetics of delipidation in various purification methods are interesting avenues for future studies.

Certainly our teabag purification may not be the only method to generate highly pure protein without the requirement for ultracentrifugation or with the potential for short purification protocols. Could resin beads be used without being confined in a teabag mesh, and simply being filtered from a crude solubilization mixture? We however foresee concerns with beads adhering to cell debris and aggregates, incomplete separation of aggregates/debris, and clogging during filtering, which all may affect yield and preparation quality. Magnetic beads have successfully been applied to capture CCR5 and CXCR4 from clarified mammalian cell lysates and subsequently reconstituted to form magnetic proteoliposomes^25,26^. Magnetic beads have further been employed for rapid screening of solubilization conditions^27^. Nevertheless, modest protein binding capacity combined with relatively high cost of magnetic beads has so far limited application for mg-scale membrane protein purification. Phase-separation represents another interesting alternative to chromatography-based methods, with a number of advantages in common with the teabag method, such as relatively simple protocols, no complex laboratory equipment is required, easily scalable and that solubilization and purification can be combined^28^. However, phase separation require typical high detergent concentrations, temperatures that may be incompatible with membrane protein stability, and protocols are highly detergent specific, necessitating optimization of the procedures for novel detergents such as maltose-neopentyl glycol amphiphiles^29^.

Transmembrane proteins are attractive drug discovery targets, but notoriously difficult to work with. In case of hAQP10 and MraY, we verified the functionality of the samples produced with teabags in separate activity assays. We demonstrated MraY enzymatic activity and its binding to the inhibitor tunicamycin using a FRET-assay and ITC experiments; similarly, we showed water and glycerol conductance across liposomes for hAQP10. The fact that it was so straightforward to apply the teabag system to purification of these proteins without further optimization—while maintaining the quality of the protein—demonstrates the strength of this method.

Collectively, we provide a package that shows the applicability of the teabag method for a variety of membrane proteins—and expression systems—including pharmaceutically relevant and challenging targets. We also provide quality control of their maintained functionality and stability. In our study, we could show that the teabag system allows a wide range of downstream applications, including biochemical and biophysical assays, ITC, X-ray crystallography and cryo-EM.

In conclusion, the teabag system provides a novel membrane protein purification method that results in the isolation of high-quality samples without the need for ultracentrifugation. The method is scalable, resin independent and allows for parallelization. The teabag system can aid membrane protein research as it allows the purification of high-quality samples in a cheap, fast, and easy way that can be used for subsequent functional and structural studies.

## Material and methods

### Construction of teabags

The teabags were prepared as described previously^17^ from a PET mesh (Sefar Petex 40 µm) using varying sizes according to the amount of affinity resin used. After filling and heat sealing, the teabags were washed with water and equilibrated with purification buffer prior to use or stored in 20% ethanol.

### *E. coli* expression and purification of MraY

The MraY protein was overexpressed according to previous methods^18^, MraY was expressed in *E. coli* BL21(gold)DE3 cells in SB medium as 500-mL cultures with 0.1 mg/mL kanamycin and 20 mM d-(+)-glucose in flasks. Cells were grown to OD_600_ = 0.7–0.9 at 37 °C and 210 rpm. Protein expression was induced with 0.1 mM IPTG at 18 °C for 17–18 h. Cells were resuspended in lysis buffer [25 mM HEPES pH 7.5, 150 mM NaCl, 10% (v/v) glycerol, 1 mM MgCl_2_, 0.1 mg/mL lysozyme, 1 mM TCEP, 1 mM PMSF, complete protease inhibitor (Roche) and 0.3 μL/mL Benzonase (Sigma)]. All further steps were carried out at 4 °C. Cells were lysed in a Constant System cell disruptor at 28 kpsi and protein was extracted from the crude lysate by addition of 40 mM dodecyl-maltoside (DDM). Insoluble fractions were removed by centrifugation. A teabag containing 3 mL cobalt Talon resin was added to the supernatant (400 mL) and incubated under gentle stirring overnight. The teabag was transferred to a 50 mL Falcon tube during each washing step for 5 min and was finally eluted in 5 CV elution buffer [20 mM HEPES pH 7.5, 150 mM NaCl, 3 mM decyl-maltoside (DM), 140 mM imidazole, 0.1 mM PMSF] for 5 min. The protein was subsequently concentrated using spin concentrators with 50 kDa cutoff and size-exclusion chromatography was carried out using a Superdex 200 10/30 column (GE Healthcare) in a mobile phase of 20 mM HEPES pH 7.5, 150 mM NaCl, 3 mM DM, 1 mM TCEP.

### Sf9 expression and purification of PAR2

PAR2 was expressed and purified according to Cheng et al.^30^. In brief, stabilized PAR2 receptor was expressed in Sf9 cells and harvested 48 h after infection. Cells were lysed in 50 mM HEPES pH 7.5, 250 mM NaCl with protease inhibitor cocktail using a Constant System cell disruptor at 25 kpsi. Membranes were collected by ultra-centrifugation and homogenized into 50 mM HEPES pH 7.5, 250 mM NaCl buffer and stored at − 80 °C. Thawed membranes were incubated with the AZ8838 ligand (final concentration 84 µM) and solubilized by the addition of 1% (w/v) lauryl maltose neopentyl glycol (LMNG)/0.1% (w/v) cholesteryl hemisuccinate (CHS) mixture. After 1.5 h, the mixture was split in three equal volumes. For one of the aliquots insoluble material was removed by ultracentrifugation for 30 min at 205,000 × *g* in a Beckman Ti45 rotor, and 0.5 mL teabags were added (Petex 40 µm mesh, Sefar 07-40/25) ~ 1 × 2 cm filled with 0.5 mL Ni-NTA resin Qiagen Superflow Material no. 1018401, lot 136232607) to the soluble fraction. The two other aliquots of membrane solubilizate were not ultracentrifuged, and 0.5 mL teabags were added directly to the membrane mixture. PAR2 protein was incubated with the teabags under extensive mixing using a magnetic stirrer for 4 h. After incubation, teabags were transferred to new tubes and washed five times with 10–15 mL washing buffer [50 mM HEPES pH 7.5, 250 mM NaCl, 0.01% (w/v) LMNG, 0.002% (w/v) CHS, 50 µM AZ8838 and 40 mM imidazole] for 5 min with rolling. PAR2 was eluted from the teabags with three consecutive 5 mL elutions with buffer [50 mM HEPES pH 7.5, 250 mM NaCl, 0.01% (w/v) LMNG, 0.002% (w/v) CHS, 50 µM AZ8838 and 300 mM imidazole] for 15 min with rolling. The eluates were concentrated to 0.5 mL using an Amicon Ultra-15 concentrator (100 kDa molecular weight cut-off). Aggregated material was removed by ultra-centrifugation at 135,520×*g* for 10 min in a Beckman Coulter bench-top centrifuge using the TLA-55 rotor. The samples were subjected to size-exclusion chromatography in 50 mM HEPES pH 7.5, 150 mM NaCl, 0.004% LMNG, 0.002% CHS, 50 μM AZ8838 or 5 μM AZ3451 on an Enrich 650 column (Bio-Rad).

### Sf9 expression and purification of KCC2

KCC2 was expressed with an N-terminal His and FLAG tag in Sf21 cells using a 20 L culture volume, and harvested 48 h after infection and frozen in − 80 °C. All further steps were carried out on ice or at 4 °C. For membrane preparation, cells were resuspended in an isotonic lysis buffer (10 mM HEPES pH 7.5, 20 mM NaCl, 1 mM KCl, 1 mM PMSF), and protease inhibitors (Roche Complete EDTA free protease inhibitor tablets). Cells were lysed by mechanical sheering using an Ultra-turrax with a 25 mm cutting head at a speed of 19,000 rpm for 2 × 20 s. Unbroken cells and debris were removed by centrifugation at 500×*g* for 15 min. Membranes were harvested by ultracentrifugation at 44,000 rpm in a Ti45 rotor (Beckman Coulter) for 30 min. Subsequently, membranes were washed with a high salt buffer (20 mM HEPES pH 7.5, 1 M NaCl and 10 mM KCl) and finally homogenized in a buffer containing 10 mM HEPES pH 7.5, 10 mM NaCl, 10 mM MgCl2, 10 mM KCl, and 40% glycerol. Membranes were flash frozen in liquid nitrogen and stored at − 80 °C until use.

KCC2 was solubilized in a buffer containing 1% (w/v) Fos-choline C14 (FC14, Anatrace #F312S), 300 mM NaCl, 480 mM KCl, 20 mM HEPES pH 7.5, 1 mM PMSF, 2 protease inhibitor tablets for 3–4 h. Four teabags, each containing 1 mL anti-FLAG M2 affinity gel, were added 15 min after initiation of solubilization. While for conventional purification 4 mL resin (anti-FLAG M2 affinity gel) was added after solubilization and separation of insoluble material by ultracentrifugation. For conventional purification, gravity flow separation was performed using Bio-Rad 7311550 polypropylene columns. The teabags were transferred to a 50 mL Falcon tube during washing in washing buffer [20 mM HEPES pH 7.5, 300 mM NaCl, 480 mM KCl, 1 mM TCEP, 0.01 mg/mL lipids (POPC:POPE:POPG, 3:1:1), 0.05% (w/v) FC14] for 15 min and was finally eluted in 3 × 1 CV wash buffer supplemented with 0.1 mg/mL FLAG-peptide (Sigma F3290-4 mg) for 15 min. The protein was subsequently concentrated using spin concentrators with 100 kDa cutoff and size-exclusion chromatography was carried out using a Superose-6 10/300 column (GE Healthcare) in a mobile phase of 20 mM HEPES pH 7.5, 300 mM NaCl, 480 mM KCl, 1 mM TCEP, 0.01 mg/mL lipids (POPC:POPE:POPG, 3:1:1), 0.05% (w/v) FC14.

Reproducibility of the teabag protocol was assessed using three technical replicates for PAR2 and KCC2; the purity of each of the three repeats was virtually identical, while the protein yield showed some variation in the range of the conventional purification protocols. For PAR2, the yield of purified protein per liter culture showed a relative standard deviation from the teabag purification of 25% compared to 18% for conventional methods, while for KCC2 these figures were 11% and 13%, respectively.

### *S. cerevisiae* expression and purification of hAQP10

The purification protocol of hAQP10 is based on work by Bjoergskov et al. and Gotfryd et al.^20,31^. The hAQP10 protein was overexpressed in *S. cerevisiae* in 6 L amino acid-supplemented minimal medium (3% glucose and 3% glycerol) with 0.1 mg/mL ampicillin and V200 (4.0 mg/L Biotin, 400 mg/L d-Pantothenic Acid, 0.4 mg/L Folic Acid, 2000 mg/L Myo-Inositol, 80 mg/L Niacin, 40 mg/L p-Aminobenzoic Acid, 80 mg/L Pyridoxin, 40 mg/L Riboflavin, 80 mg/L Thiamin) diluted 200 times in the flasks. Cells were grown to OD_600_ = 1.0–1.5 at 30 °C and 100 rpm. Protein expression was induced with 2% galactose at 15 °C for 24 h, then harvested and placed at − 80 °C to facilitate cell lysis. All further steps were carried out at 4 °C. Cells were resuspended in 25 mM Tris–HCl pH 7.5, 500 mM NaCl, 20% v/v glycerol, 5 mM BME, 1 mM PMSF and LPC protease inhibitors, and lysed using a bead beating system with glass beads. The cell suspension was first separated using 3000 rpm where the lysate was further spun down at 190,000×*g* for 3 h in a Beckman Ti45 rotor. The membranes were resuspended in 20 mM Tris–HCl pH 7.5, 200 mM NaCl, 20% v/v glycerol, 5 mM BME, 1 mM PMSF and LPC protease inhibitors at 0.2 g membranes per mL (buffer), and solubilized in 2% w/v DM (Anatrace, Maumee, OH, USA) for 4 h. The membrane suspension was diluted to 1% DM. Two teabags containing in total 5 mL Ni–NTA resin were added to the supernatant and incubated under gentle stirring for 4 h. The teabags were transferred to a 50 mL Falcon tube during washing in washing buffer (20 mM Tris–HCl pH 7.5, 200 mM NaCl, 20% v/v glycerol, 5 mM BME, 50 mM imidazole pH 7.5, 0.2% DM) for 5 min, and was finally eluted in 5 CV elution buffer (20 mM Tris–HCl pH 7.5, 200 mM NaCl, 20% v/v glycerol, 5 mM BME, 500 mM imidazole pH 7.5, 0.2% DM) for 5 min. The protein was subsequently concentrated using spin concentrators with 100 kDa cutoff and size-exclusion chromatography was carried out using a Superdex 200 increase 10/300 column (GE Healthcare) in a mobile phase of 20 mM Tris–HCl pH 7.5, 100 mM NaCl, 10% v/v glycerol, 2 mM BME, 0.4% Nonyl Glucopyranoside (NG) (Anatrace, Maumee, OH, USA).

### *S. cerevisiae* expression and purification of hClC-1

The purification protocol for ClC-1 is similar to the one of hAQP10 and based on previous work by Wang et al.^21^. E232A ClC-1 was also overexpressed in *S. cerevisiae* in 2 L minimal medium in a bioreactor with 0.1 mg/mL ampicillin and V200 (see hAQP10 methods). Cells were grown until stagnation. Protein expression was induced with 2% galactose at 15 °C for 48 h then harvested. All further steps were carried out at 4 °C. Cells were resuspended in 25 mM imidazole pH 7.5, 1 mM EGTA, 1 mM EDTA, 10% v/v glycerol, 5 mM BME, 1 mM PMSF and LPC protease inhibitors and lysed using a bead beating system with glass beads. The cell suspension was first separated using 1000×*g* for 10 min where the lysate was further spun down at 190,000×*g* for 90 min in a Beckman Ti45 rotor. The membranes were resuspended in 50 mM Tris–HCl pH 7.5, 300 mM NaCl, 10% v/v glycerol, 5 mM BME, 1 mM PMSF and LPC protease inhibitors, and solubilized in 1% w/v DDM and 0.33% CHS (Anatrace, Maumee, OH, USA) for 4 h. Two teabags containing in total 5 mL Ni–NTA resin were added to the supernatant and incubated under gentle stirring for 4 h after addition of 30 mM imidazole pH 7.5. The teabags were transferred to a 50 mL Falcon tube during washing with high salt buffer (50 mM Tris–HCl pH 7.5, 800 mM NaCl, 5% v/v glycerol, 5 mM BME, 0.4 mg/mL DDM and 0.04 mg/mL CHS) for 5 min, followed by washing with low salt buffer (50 mM Tris–HCl pH 7.5, 300 mM NaCl, 5% v/v glycerol, 5 mM BME, 0.4 mg/mL DDM and 0.04 mg/mL CHS) for 5 min. Subsequently, the protein was eluted using 5 CV elution buffer (50 mM Tris–HCl pH 7.5, 300 mM NaCl, 5% v/v glycerol, 5 mM BME, 0.4 mg/mL DDM and 0.04 mg/mL CHS, 200 mM imidazole pH 7.5). The top fractions were subsequently concentrated using spin concentrators with 100 kDa cutoff to 1 mL. Amphipols PMAL-8 was then added in a 1:5 w/w ratio and incubated overnight. Next, the sample was dialyzed overnight in a Slide-A-Lyzer, 20 K MWCO, (Pierce, USA) against 0.2 g Bio-Beads (Bio-Rad) to remove detergents and 40 mL of the final cryo buffer (20 mM Tris–HCl pH 7.5, 100 mM NaCl, 0.2 mM TCEP). Size-exclusion chromatography was carried out using a Superdex 200 increase 10/300 column (GE Healthcare) in a mobile phase of 20 mM Tris–HCl pH 7.5, 100 mM NaCl and 0.2 mM TCEP. The sample was concentrated to 0.7 mg/mL using a VivaSpin 100 MWCO (Sartorius, Germany) for negative staining. Purified ClC-1 solutions were diluted to10 μg/mL with the same buffer as for gel filtration. 3.5 μL diluted sample were applied to glow-discharged, 400 mesh carbon-coated grids (Tem-Sem, China) and incubated for 1 min. The grids were washed twice with 4 μL of staining solution (2% uranyl acetate) and then stained with 4 μL of staining solution for 1 min. Negative stain micrographs were taken on a transmission electron microscope operated at 100 kV (Philips CM 100) at a magnification of 69,000.

### Functional assay MraY

Activity of MraY was investigated using a previously published FRET-based assay^19^. Briefly, the assay was performed in 384 well black polystyrene assay plates in a total volume of 9 μL. An assay buffer containing 50 mM Tris–HCl pH 7.5, 0.5 M trehalose, 150 mM KCl, 50 mM MgCl_2_, 1 mM DTT, 0.05% Triton X-100 was used. 122 nM purified MraY protein was incubated with 20 μM undecaprenyl phosphate (C55P) and 24 μM 1,2-dipalmitoyl-sn-glycero-3-phosphoethanolamine-*N*-(lissamine rhodamine B sulfonyl) (LRPE) in assay buffer for 30 min. The reaction was initiated with 2 μM UDP–MurNAc-l-Ala-γ-D-Glu-*m*-DAP-D-Ala-D-Ala labeled with BODIPY–FL–sulfosuccinimidyl ester (B-UNAM-pp) and 0 mM or 5 mM uridine 5′-monophosphate (UMP). Fluorescence was excited at 485 nm and detected simultaneously at 520 nm and 590 nm every minute for 30 min in a PheraStar plate reader (BMG Labtech, Cary, NC, USA).

The ITC titration experiments were conducted on a MicroCal Auto-200 system (GE Healthcare) using 15 µM MraY in a buffer containing 20 mM HEPES pH 7.5, 150 mM NaCl, 3 mM DM and 1 mM TCEP. Tunicamycin (200 µM) was added by injecting 13 × 3 µL at 25 °C with 150 s waiting time between subsequent injections. All measurements were performed in triplicates and the K_d_ value is 233 ± 30 nM.

Differential scanning fluorimetry (DSF) measurements were performed on a Prometheus NT.48 (NanoTemper Technologies). MraY protein samples were prepared at a concentration of 0.5 mg/mL protein and 50 µM compound (or 5% DMSO as a control) in a buffer containing 20 mM HEPES pH 7.5, 150 mM NaCl, 3 mM DM, 1 mM TCEP. Technical duplicates were prepared and loaded in glass capillaries that were positioned on the deck of the DSF instrument for analysis, each experiment was conducted two times. Melting curves were performed from 20 to 95 °C with a ramping of 0.5 °C/min at 60% excitation power. The PR.ThermControl software was used to perform the experiment and to analyze the data. To determine Tm_1/2,_ the maximum of the first derivative of the 330 nm wavelength was used.

### Functional assay AQP10

For liposome production *E. coli* polar lipid extract (Avanti polar lipids) were dissolved in chloroform in a glass vial to a concentration of 25 mg/mL, followed by removal of the chloroform using a light stream of nitrogen gas. The vial with the resulting lipid film was then placed in a desiccator overnight. The lipid film from the glass vial was rehydrated to a lipid concentration of 20 mg/mL using reconstitution buffer (20 mM Tris–HCl pH 7.5, 200 mM NaCl) with 10 mM fluorophore ((5)6-carboxyfluorescein (Sigma)) added. The resuspended lipid solution was sonicated using a bath sonicator for 3 × 15 min. Next, the lipid solution was flash-frozen 3 times in liquid nitrogen. Once thawed at room temperature, the lipid solution was passed through a 200 nm polycarbonate filter 11 times, using an extruder (Mini-Extruder, Avanti). Subsequently, the lipids were diluted to 4 mg/mL using reconstitution buffer with 25% glycerol, 0.4% NG and 0.02% Triton X-100 added. The purified hAQP10 was added to the lipid solution using a lipid-to-protein ratio of 25 (weight:weight). The sample was dialyzed overnight at 4 °C against reconstitution buffer. The samples were centrifuged at 57,000×*g* (1.5 h), and the resulting pellets were dissolved in resuspension buffer (20 mM Tris–HCl pH 7.4, 200 mM NaCl and for the glycerol measurements 500 mOsm glycerol was added to the resuspension buffer). The stopped-flow experiments were performed using an SX-20 Stopped-Flow Spectrometer system (Applied Photophysics) in which the liposomes were rapidly mixed with a reaction buffer (resuspension buffer with 500 mOsm Sucrose added). The data were collected with the excitation wavelength of 495 nm, and measured for 5 s for water flux measurements and 10 s for glycerol flux. All data were collected at room temperature. Empty liposomes were used as a negative control and their flux rates subtracted from the flux rates of the AQP10 containing liposomes. The data were analyzed and plotted using GraphPad Prism 8. Each sample was measured 10 times, then averaged and normalized.

### Surface plasmon resonance

Measurements were performed on a BIAcore S200 (GE healthcare) at 20 °C using running buffer; 20 mM HEPES, 480 mM KCl, 300 mM NaCl, 1 mM TCEP, 0.05% FC14, 10 mg/L lipids (POPC:POPE:POPG, 3:1:1), 0.5% DMSO. Purified KCC2 was covalently immobilized on EDC/NHS activated NID500L chip (Xantec) utilizing the 6His-tag for pre-concentration of protein on the chip to a final level of 3000 ± 500 RU. Concentration series of compounds (n = 3) were dispensed using a Digital dispenser (Tecan), normalized to 0.5% DMSO and injected at 30 μL/min for 1 min. Binding levels were fitted to a Langmuir 1:1 interaction model to extract steady state affinity (K_d_).

### Crystallization

hAQP10 crystals were produced by hanging-drop vapor diffusion at 18 °C by mixing protein solution (~ 4 mg/ mL) with a reservoir solution (100 mM MES-monohydrate-NaOH pH 6.0, 19% PEG 2K MME, 5% glycerol) in a 1:1 ratio. The crystals appeared after 1–2 days and were harvested followed by flash freezing in liquid nitrogen. X-ray diffraction data were collected using an EIGER detector at the Paul Scherrer Institut, Villigen, Switzerland, beam line X06SA.

## Supplementary information

Supplementary Information
