# Supplementary material for: The rapid “teabag” method for high-end purification of membrane proteins
Source: Sci Rep. 2020 Sep 30;10:16167. doi: 10.1038/s41598-020-73285-9 (PMC7528119; doi:10.1038/s41598-020-73285-9)
Supplement: Supplementary file 1 — Supplementary Information [file 41598_2020_73285_MOESM1_ESM.docx]

**Supplementary figures**

**The rapid “teabag” method for high-end purification of membrane proteins**

**Authors**

Jenny Hering^1,2#^, Julie Winkel Missel^3#^, Liying Zhang^3^, Anders Gunnarsson^1^, Marie Castaldo^4^, Per Amstrup Pedersen^5^, Margareta Ek^1^, Pontus Gourdon^3, 6^, Harm Jan Snijder^4*^

^1^ Structure, Biophysics & FBLG, Discovery Sciences, R&D, AstraZeneca, Gothenburg, Sweden

^2^ Department of Chemistry and Molecular Biology, University of Gothenburg, Sweden

^3^ Department of Biomedical Sciences, University of Copenhagen, Denmark

^4^ Discovery Biology, Discovery Sciences, R&D, AstraZeneca, Gothenburg, Sweden

^5^ Department of Biology, University of Copenhagen, Denmark

^6^ Department of Experimental Medical Science, Lund University, Sweden

^#^ Both authors contributed equally to this work

* To whom correspondence should be addressed: Arjan (Harm Jan) Snijder, Discovery Sciences, R&D, AstraZeneca, Gothenburg, Sweden; e-mail: arjan.snijder@astrazeneca.com

**
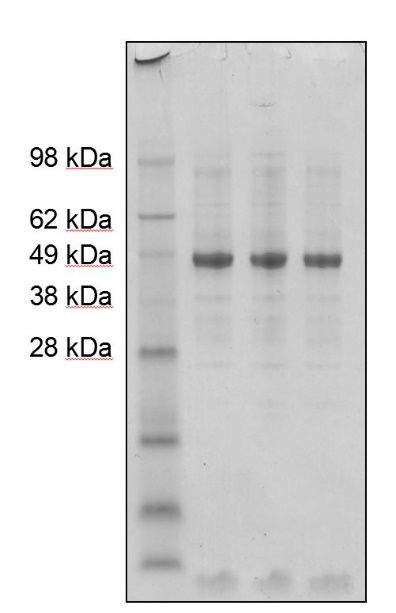
**

**Supplementary figure 1.** SDS-PAGE analysis of 2 µg PAR2 purified by teabag Ni-IMAC purification and size-exclusion chromatography. Lanes 1 and 2 are two identical experiments where teabags were incubated in the crude membrane detergent mix, lane 3 represents an experiment where the insoluble material is first removed by ultracentrifugation prior to teabag purification.


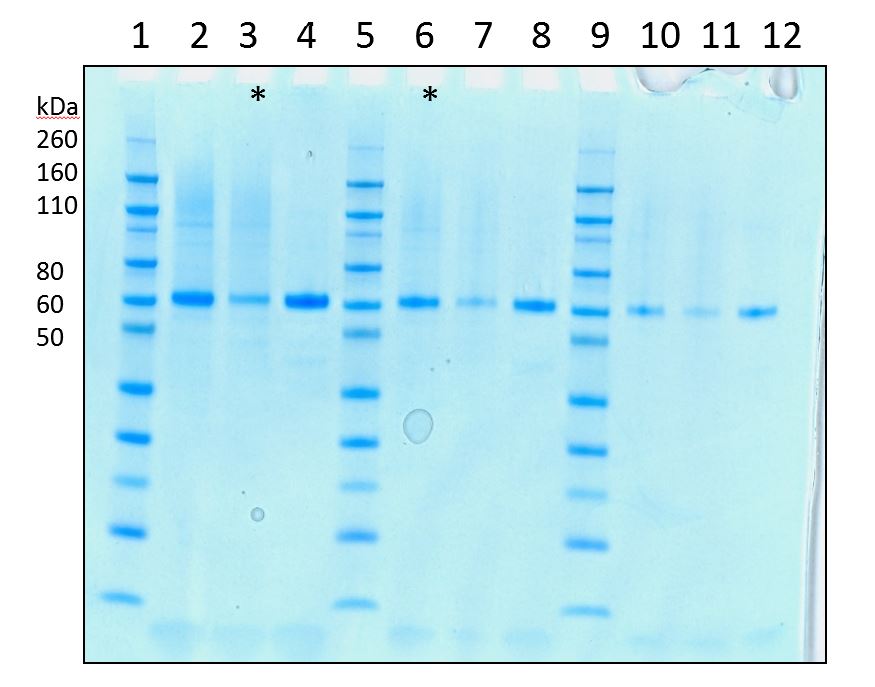


**Supplementary figure 2.** Two-fold serial dilutions of PAR2 protein are analyzed on SDS-PAGE. Conventional purification is shown in lane 2, 6 and 10, starting from 2.5 µg protein in lane 2. Teabag purification is presented in lane 3, 7 and 11, with 1.2 µg protein in lane 3. Lanes 4, 8 and 12 sample not relevant in the context of this paper. Asterixis indicate the lanes shown in figure 2 of the main paper. Lanes 1, 5 and 9 marker.


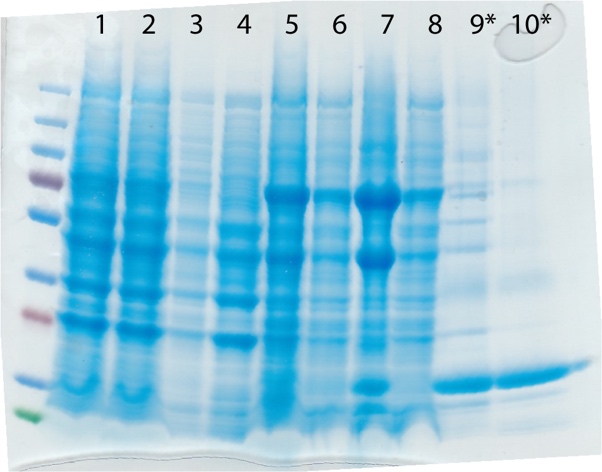


**Supplementary figure 3.** SDS-PAGE analysis of a typical AQP10 affinity purification and comparison with teabag purification. Lane 1 and 2 are solubilized membranes, lanes 3 and 4 show flow though fractions, lanes 9 and 10 IMAC purified AQP10, respectively conventional and teabag purification. Lanes 5-8 are samples unrelated to this paper. Asterixis indicate the lanes shown in figure 2 of the main paper.


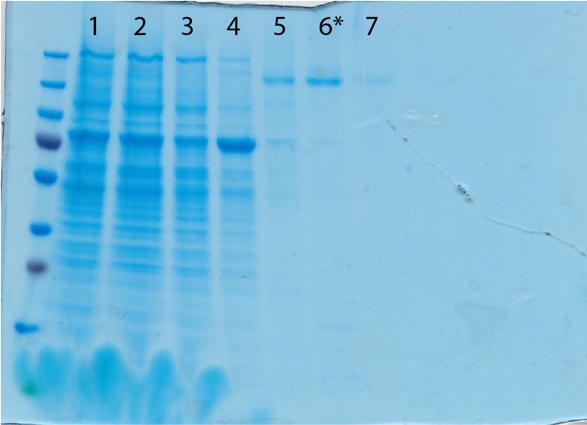


**Supplementary figure 4a.** SDS-PAGE analysis of a conventional affinity purification of CLC-1. Lane 1 total membrane fraction, lane 2 solubilized material after centrifugation, lane 3 IMAC flow through fraction, lane 4 IMAC wash one, lane 5 IMAC wash two, lane 6 IMAC elution and finally lane 7 IMAC elution fraction 2. Lane 6 indicated with an asterix is shown in figure 2 of the main paper.


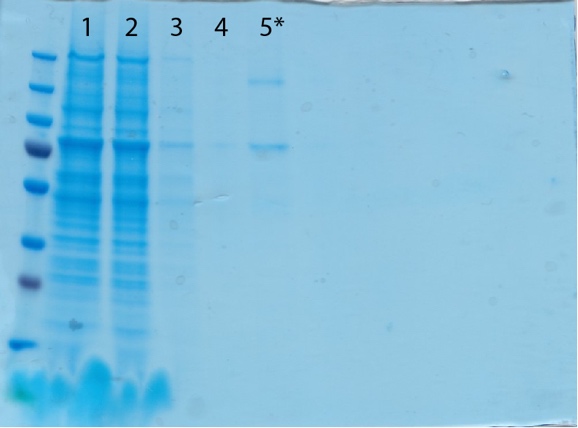


**Supplementary figure 4b.** SDS-PAGE analysis of a teabag purification of CLC-1. Lane 1 total membrane fraction, lane 2 solubilized material after teabag incubation, lane 3 IMAC wash one, lane 4 IMAC wash two, lane 5 IMAC elution as shown in figure 2 of the main paper.


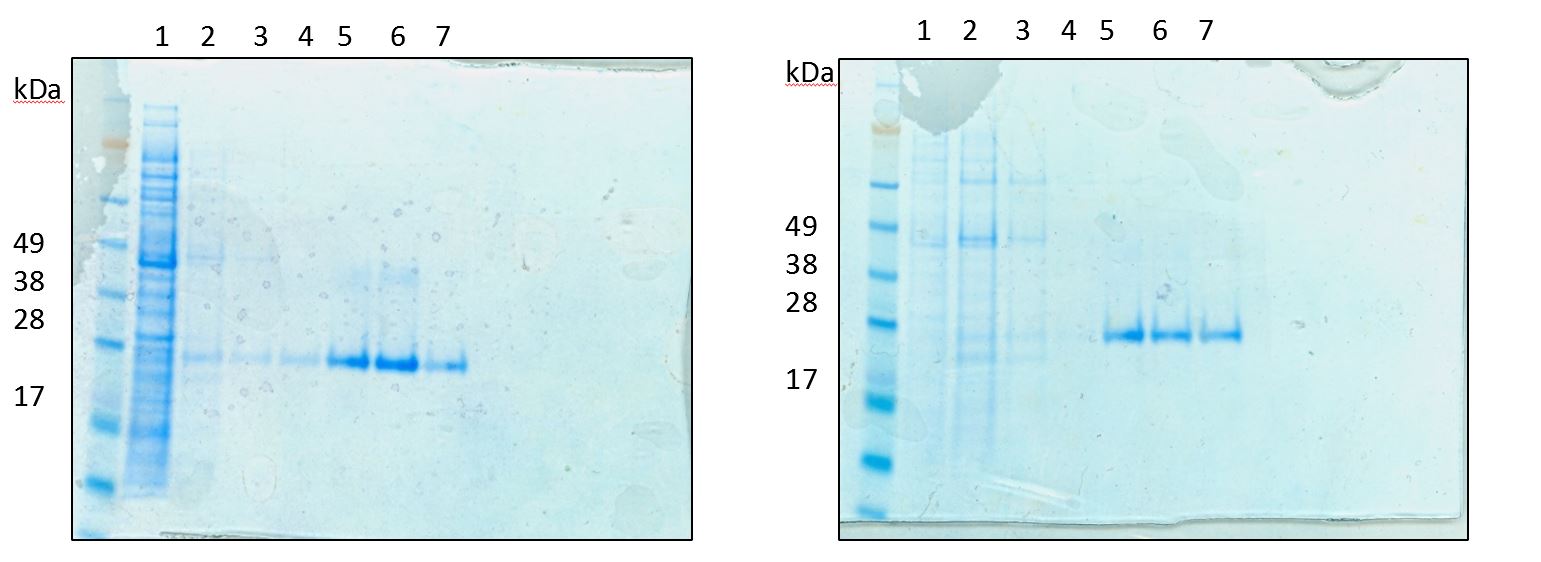


**Supplementary figure 5a and b**. Conventional column purification (left) versus teabag purification (right). Lane 1-4 are consecutive washing fractions, lanes 5-7 are elution fractions as show in figure 2 of the main paper.


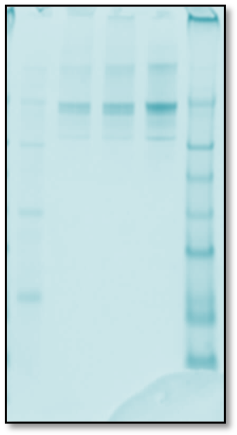

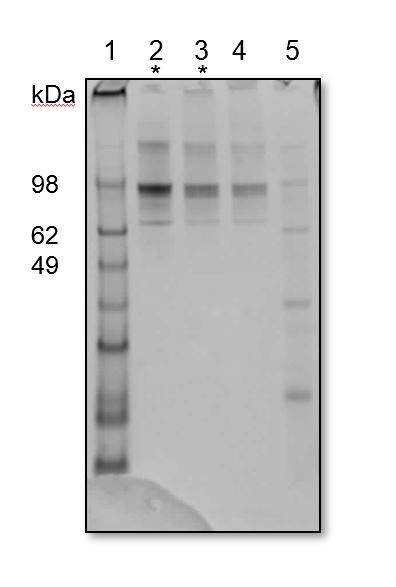


**Supplementary figure 6**. SDS-PAGE analysis of side-by-side teabag and conventional purification of KCC2. Lane 1 marker, lane 2 teabag purification, lane 3 conventional purification, lane 4 teabag purification from other occasion. Lane 5 irrelevant sample. Lanes indicated by asterixis are shown in figure 2 of the main text.
